# The relative contribution of intervertebral disc and vertebral wedging to the development of thoracic kyphosis and lumbar lordosis in asymptomatic adolescents

**DOI:** 10.1186/1748-7161-10-S1-P16

**Published:** 2015-01-19

**Authors:** Wen Zhang, Zhen Liu, Shi Lin, Winnie Chu, Tsz-ping Lam, Zezhang Zhu, Jack CY Cheng, Yong Qiu

**Affiliations:** 1 Spine Surgery, Affiliated Drum Tower Hospital of Nanjing University Medical School, Nanjing 210008, China; 2 Department of Orthopaedics and Traumatology, Chinese University of Hong Kong, Hong Kong, China; 3 Joint Scoliosis Research Center of the Chinese University of Hong Kong & Nanjing University, Nanjing, China; 4 Department of Radiology, Chinese University of Hong Kong, Hong Kong, China

## Objective

To analyze the contribution of the intervertebral disc and vertebral wedging to thoracic kyphosis and lumbar lordosis in different growth stages of asymptomatic adolescence.

## Methods and materials

A retrospective study of 170 asymptomatic adolescent volunteers was performed and all of them had the standing whole-spine lateral radiography. All subjects were divided into 3 groups by age: Group A (10-12 years), Group B (13-15 years), and Group C (16-18 years). Four different radiographic parameters were measured: lumbar lordosis (LL, L1-L5), thoracic kyphosis (TK, T4-T12), wedging angle of each disc and vertebra between T4 and L5. In addition, the percent of the sum of T4-T12 disc/vertebral wedging angle in the TK and the sum of the L1-L5 disc/vertebral wedging angle in the LL were calculated.

## Results

The LL increased by 5.1° from group A to Group C (P=0.028), followed by an increasing contribution of the lumbar disc wedging from 90.0 percent in Group A to 96.4 percent in Group C (P=0.025) and a decreasing contribution of the lumbar vertebral wedging from 9.0% in Group A to 2.9 percent in Group C (P=0.02). As for the thoracic spine, the TK increased by 5.8° from Group A to Group C (p=0.021), but no significant differences in the contribution of both disc and vertebral wedging to TK were found among the three groups.

## Conclusions

In the different growth stages of asymptomatic adolescences, an increasing contribution of the disc wedging and a decreasing of the vertebral wedging to the LL are results of age-associated process. However, the contribution of the disc and vertebral wedging to the TK remains stable in different growth periods.

